# *Veronica officinalis* Product Authentication Using DNA Metabarcoding and HPLC-MS Reveals Widespread Adulteration with *Veronica chamaedrys*

**DOI:** 10.3389/fphar.2017.00378

**Published:** 2017-06-19

**Authors:** Ancuta C. Raclariu, Andrei Mocan, Madalina O. Popa, Laurian Vlase, Mihael C. Ichim, Gianina Crisan, Anne K. Brysting, Hugo de Boer

**Affiliations:** ^1^Plant Evolution and Metabarcoding Group, Natural History Museum, University of Oslo, Oslo, Norway; ^2^Stejarul Research Centre for Biological Sciences, National Institute of Research and Development for Biological Sciences (NIRDBS), Piatra Neamţ, Romania; ^3^Department of Pharmaceutical Botany, Faculty of Pharmacy, Iuliu Hatieganu University of Medicine and Pharmacy, Cluj-Napoca, Romania; ^4^ICHAT and Institute for Life Sciences, University of Agricultural Sciences and Veterinary Medicine of Cluj-Napoca, Cluj-Napoca, Romania; ^5^Department of Pharmaceutical Technology and Biopharmaceutics, Iuliu Hatieganu University of Medicine and Pharmacy, Cluj-Napoca, Romania; ^6^Department of Biosciences, Centre for Ecological and Evolutionary Synthesis (CEES), University of Oslo, Oslo, Norway; ^7^Department of Organismal Biology, Evolutionary Biology Centre, Uppsala University, Uppsala, Sweden

**Keywords:** adulteration, DNA metabarcoding, herbal products, HPLC-MS, *Veronica chamaedrys*, *Veronica officinalis*

## Abstract

Studying herbal products derived from local and traditional knowledge and their value chains is one of the main challenges in ethnopharmacology. The majority of these products have a long history of use, but non-harmonized trade and differences in regulatory policies between countries impact their value chains and lead to concerns over product efficacy, safety and quality. *Veronica officinalis* L. (common speedwell), a member of Plantaginaceae family, has a long history of use in European traditional medicine, mainly in central eastern Europe and the Balkans. However, no specified control tests are available either to establish the quality of derived herbal products or for the discrimination of its most common substitute, *V. chamaedrys* L. (germander speedwell). In this study, we use DNA metabarcoding and high performance liquid chromatography coupled with mass spectrometry (HPLC-MS) to authenticate sixteen *V. officinalis* herbal products and compare the potential of the two approaches to detect substitution, adulteration and the use of unreported constituents. HPLC-MS showed high resolution in detecting phytochemical target compounds, but did not enable detection of specific plant species in the products. DNA metabarcoding detected *V. officinalis* in only 15% of the products, whereas it detected *V. chamaedrys* in 62% of the products. The results confirm that DNA metabarcoding can be used to test for the presence of *Veronica* species, and detect substitution and/or admixture of other *Veronica* species, as well as simultaneously detect all other species present. Our results confirm that none of the herbal products contained exactly the species listed on the label, and all included substitutes, contaminants or fillers. This study highlights the need for authentication of raw herbals along the value chain of these products. An integrative methodology can assess both the quality of herbal products in terms of target compound concentrations and species composition, as well as admixture and substitution with other chemical compounds and plants.

## Introduction

Traditional herbal medicines play an important role in meeting healthcare needs around the world, and complementary and alternative medicines based on these are gaining in importance in many industrialized countries as a perceived healthy alternative to synthetic pharmaceuticals (World Health Organization, 2013). The commercialization of such products is undergoing a transition process from unregulated locally traded products to internationally traded mass produced herbal pharmaceuticals and food supplements (Heinrich, 2010; Leonti and Casu, 2013). A challenge in ethnopharmacology is to study such novel herbal products and their value chains (Heinrich, 2010; Atanasov et al., 2015), as these are impacted by non-harmonized regulatory and trade policies (Booker et al., 2012, 2016).

The regulation of herbal products varies globally and between EU member countries, these falling under specific legislation depending on their intended use, for instance, as food, food supplements, herbal medicines, or homeopathic products. Similar to the United States and Canada, the EU currently does not have a centralized marketing authorization procedure for herbal products, but instead provides relevant methodological specifications to guarantee manufacturing quality. The EU also lacks a central public institution for pharmacovigilance of the herbal products, and the primary legal responsibility for safety of the marketed herbal products is delegated to the pharmaceutical industry, whereas national pharmacovigilance agencies monitor drug safety.

A lack of standards for studies of medicinal plants has been previously identified (cf. Ríos and Recio, 2005; Gertsch, 2009) and several authors have advocated a stronger guidance of value chains to mitigate this shortcoming (Rios et al., 1988; Cos et al., 2006; Verpoorte, 2008). Current quality assessment methods rely mainly on morphology and analytical chemistry based methods detailed in national or EU pharmacopoeias (EDQM - Council of Europe, 2014). However, the resolution of these methods is highly influenced by factors such as the phenotypic plasticity, morphological cryptic taxa, species-specific chemical markers, growing conditions, harvesting process, storage condition, or the extraction procedure (Barnes, 2003; Bickford et al., 2007; Jiang et al., 2010; Shaw et al., 2012).

In addition to ensuring that herbal products contain the intended ingredients in the right quantities, an important problem is the use of substitutes and undeclared fillers (Coghlan et al., 2012, 2015; Newmaster et al., 2013). Misidentification (Saslis-Lagoudakis et al., 2015) or incongruences in the vernacular names of the raw material (Farah et al., 2006; Ouarghidi et al., 2012; Walker and Applequist, 2012; De Boer et al., 2015) can lead to accidental substitution, but rising prices of raw materials and the high demand for such products also provide an incentive for deliberate adulteration. The use of unlabelled ingredients presents a serious safety challenge as adverse drug reactions cannot be associated to the product label and ingredients (Heubl, 2010; Gilbert, 2011). Recent studies of herbal medicine and herbal food supplement value chains using a combination of phytochemical and metabolomics analyses have found that adulteration of these products often occurs in the first stages of their value chains (Booker et al., 2012, 2014, 2016).

*Veronica officinalis* L. (common speedwell) is a popular medicinal plant in several European countries, especially in central eastern Europe and the Balkans, and used in herbal medicine both traditionally and increasingly in commercial herbal products. *Veronica* L. (Plantaginaceae) is a genus of about 450 species (Albach and Meudt, 2010) occurring over most of the Northern Hemisphere and in many parts of the Southern Hemisphere (Mabberley, 2008). Modern systematic studies of the genus have focused on molecular phylogenies of several nuclear and plastid markers: the nuclear ribosomal internal transcribed spacer, nrITS (Wagstaff and Garnock-Jones, 1998; Albach and Chase, 2001; Albach et al., 2004; Buhk et al., 2015), the plastid regions *trn*L-F (Albach et al., 2004, 2006; Buhk et al., 2015), *ndh*F-*rpl*32 (Sonibare et al., 2014; Albach and Al-Gharaibeh, 2015; Buhk et al., 2015), *trn*H-*psb*A (Sonibare et al., 2014; Albach and Al-Gharaibeh, 2015), *rps*16 intron (Albach and Chase, 2004), *rps*16–*trn*K (Sonibare et al., 2014; Albach and Al-Gharaibeh, 2015).

### Ethnopharmacological Importance of *Veronica* Species

Several *Veronica* species are used in European traditional medicine to treat respiratory affections, as an expectorant, an antiscorbutic, a diuretic, and for wound healing (Baytop, 1984; Harput et al., 2002a). In Turkey, the aerial parts of *Veronica anagallis-aquatica* L. are boiled in milk to obtain a poultice used to alleviate abdominal pain (Küpeli et al., 2005), or used as a bath cure to relieve rheumatic pain (Fujita et al., 1995). In the Valdesi valley of Italy, in the poorer villages *Veronica* species are traditionally used as an inexpensive substitute for imported teas and this has been posed as an explanation of the modern use of *Veronica* species in recreational teas (Bellia and Pieroni, 2015). In traditional Chinese medicine *V. anagallis-aquatica* is used for the treatment of influenza, hemoptysis, laryngopharyngitis, and hernia (Su et al., 1999). In Europe, *V. officinalis* is the best-known and most commonly used species (**Table 1**). The main ethnopharmacological indications for *V. officinalis* are inflammatory conditions, rheumatism, stomach ulcers, respiratory ailments (Scarlat et al., 1985; Mocan et al., 2015b), and nervous, cardiovascular and metabolism system disorders (Vogl et al., 2013).

**Table 1:** Traditional uses of *Veronica officinalis* in different European countries.

Country	Use	Reference
Austria	Cardiovascular system	Vogl et al., 2013
	Metabolism	
	Nervous system	
	Respiratory tract	
Bulgaria	Appetizer	Ivancheva and Stantcheva, 2000; Leporatti and Ivancheva, 2003


	Anti-inflammatory	
	Antitussive	
	Asthma	
	Coughs	
	Expectorant	
	Pharyngitis	
	Tonsillitis	
Italy	Recreational tea	Bellia and Pieroni, 2015
Montenegro	Bronchitis	Menković et al., 2011
	Rheumatic complaints	
	Skin diseases	
	Wounds	
Romania	Antiulcerous activity	Tamas et al., 1984; Scarlat et al., 1985; Crişan et al., 2007; Gründemann et al., 2013
	Catarrh	
	Cough	
	Hepatoprotective activity	
	Hypocholesterolemic effect	
	Kidney diseases	
	Lung diseases	
	Wound healing properties	
Serbia	Against anemia	Zlatković et al., 2014
	Hypolipemic	
	Treatment of skin diseases	
Sweden	Recreational tea	Sõukand et al., 2013


### Phytochemical and Pharmacological Studies

*Veronica* species are rich in iridoid glucosides, mainly aucubin, catalpol and benzoic and cinnamic acid esters of catalpol, all compounds that have previously been investigated for their bioactivities (Harput et al., 2002a,b, 2014; Saracoglu et al., 2002). Saracoglu and Harput (2012) showed that some iridoid glucosides of *V. anagallis-aquatica*, *V. persica* Poir, and *V. thymoides* P.H.Davis subsp. *pseudocinerea* M.A.Fisch. exhibited cytostatic and cytotoxic activities on human and murine cancer cell lines, with verminoside being the most cytotoxic compound and Harput et al. (2002a) showed that *V. cymbalaria* Bodard, *V. hederifolia* L*., V. pectinata* L. var. *glandulosa*, *V. persica* and *V. polita* Fr. possess anti-inflammatory, cytotoxic and radical scavenging activities. Vogl et al. (2013) showed for the first time *in vitro* anti-inflammatory activity of the extracts prepared from *V. officinalis*. Nikolova (2011) found that *V. bellidioides* L., *V. kellereri* Degen & Urum., *V. vindobonensis* (M.A.Fisch.) M.A.Fisch., *V. beccabunga* L., *V. rhodopea* Degen ex Stoj. & Stef. and *V. austriaca* L. showed significant antioxidant properties.

### Quality Issues of *Veronica officinalis* Herbal Products

As a folk remedy, *V. officinalis* is used mostly in traditional medicine, and no current national pharmacopeia monographs include standards for quality control or authentication of *Veronica* herbal products. Identification tests currently available to detect substitutes and adulterants rely on chromatographic methods, usually as a combination of separate approaches (e.g., HPLC with TLC-densitometry) or a combination of different approaches into a single procedure (e.g., HPLC-UV, HPLC-MS or GC-MC) (European Medicine Agency [EMA], 2006; EDQM - Council of Europe, 2014).

All raw materials of *V. officinalis* used in the marketed herbal products are harvested from the spontaneous flora, and sourced from several countries in central eastern Europe and the Balkans. Kathe et al. (2003) and Ichim et al. (2011) cite that the exploitation pressure of wild-harvesting could potentially threaten the species. The limited availability of *V. officinalis* is a likely driver behind the widespread substitution with *V. chamaedrys* L. (germander speedwell), an abundant herb of grassland and forest edges, with no proven therapeutic uses Crişan et al. (2007, 2011). Furthermore, even though their vegetative morphology is clearly distinct, the flowers of *V. officinalis* and *V. chamaedrys* can easily be confused (Leyel, 1937). Crişan et al. (2001, 2011) report histological, anatomical and phytochemical characters that distinguish *V. officinalis* and *V. chamaedrys* and propose a rapid differentiation method using thin-layer chromatography (TLC) of species-specific phenyl-propanoic compounds. These analytical methods enable chemical identification of some target compounds, but cannot rule out that other *Veronica* species, or mixtures thereof, might be present in the investigated products. Marketed herbal products are complex formulations that are usually highly processed and consist of numerous ingredients.

### DNA Barcoding and Metabarcoding

Rapid advances in DNA sequencing have enabled cost-effective use of DNA sequences for species identification. Hebert et al. (2003) coined the concept of DNA barcoding as a rapid and accurate way of species-level identifications using short standard DNA regions. Different combinations of plastid markers (e.g., rbcL, matK, and trnH-psbA) and the nuclear ribosomal internal transcribed spacer (nrITS) have been proposed as the primary barcodes for plants, and the current core barcodes are rbcL and matK, supplemented by nrITS (Kress and Erickson, 2007; CBOL Plant Working Group, 2009; China Plant BOL Group, 2011; Hollingsworth et al., 2011). Several studies have shown that about 75–85%, and in some floras over 90%, of plant species can be identified to species level using DNA barcoding (Lahaye et al., 2008; Kress et al., 2009, 2010; Chen et al., 2010; Burgess et al., 2011). In taxonomically difficult plant groups, where hybridization is frequent, or in lineages of relatively young age the use of traditional barcodes may not be reliable (Kress and Erickson, 2007; Fazekas et al., 2009). In such groups the use of low-coverage shotgun sequencing of genomic DNA could resolve relationships and augment current plant barcodes (Coissac et al., 2016; Hollingsworth et al., 2016).

Techen et al. (2014) reviewed the progress made in DNA barcoding of medicinal plants. DNA barcoding of herbal products has revealed alarming levels of substitution within marketed herbal products, for instance, 6% in saw palmetto herbal dietary supplements (Little and Jeanson, 2013), 16% in ginkgo products (Little, 2014), 25% in black cohosh (Baker et al., 2012), 33% in herbal teas (Stoeckle et al., 2011), 50% in ginseng (Wallace et al., 2012), 37% in Senna and 50% in Cassia market products (Seethapathy et al., 2014). Newmaster et al. (2013) tested 44 herbal products sold in North America using DNA barcoding and found that 59% contained species not listed on the label, and only 2 out of 12 screened companies had products free of substitution, contamination or unreported fillers. DNA metabarcoding, a combination of high-throughput sequencing (HTS) and polymerase chain reaction based DNA amplification, provides simultaneous taxonomic identification of taxa from samples containing DNA from different origins (Taberlet et al., 2012). DNA metabarcoding studies are providing insights into species composition of complex mixtures of DNA such as processed herbal products. For example, Coghlan et al. (2012) evaluated the species composition of fifteen highly processed traditional Chinese medicines (TCM) and found that these contained species and genera included on CITES appendices I and II. Similarly, Cheng et al. (2014) found a high level of contamination within 27 investigated herbal preparations, Ivanova et al. (2016) found that all 15 tested herbal supplements contained species not listed on the product label, and Raclariu et al. (2017) authenticated 78 *Hypericum perforatum* herbal products and found the target species in only 68% products while detecting incongruence between constituent species and those listed on the label in all products.

In this study we test the hypothesis that dwindling availability of wild *V. officinalis* has resulted in admixture of *V. chamaedrys* and other *Veronica* species in *Veronica* herbal products. Substitution of *V. officinalis* with *V. chamaedrys* would not only be fraudulent from a commercial perspective, but would also leave anticipating consumers with a herbal product without proven therapeutic activity. We approach this hypothesis using DNA metabarcoding and HPLC-MS to authenticate and detect species diversity in European *Veronica* herbal products, and aim to answer the following research questions: (1) Can HPLC-MS be used to distinguish *V. officinalis* from *V. chamaedrys* and to identify exclusive presence of *V. officinalis* in herbal products?; (2) Can DNA metabarcoding be used to test for the presence of *V. officinalis* in herbal products, to detect substitution and adulteration for other *Veronica* species and/or presence of other off label plant species?

## Materials and Methods

### Sample Collection

Sixteen single and multi-ingredient herbal products including *V. officinalis* on the label were purchased from retail stores and pharmacies (14) and via e-commerce (2). The products included herbal teas (12), extracts (2), capsules (1), and candies (1). An overview of the samples is included in the Supplementary Table S1, but identifying information on the producer/importer, product name, lot number, expiration date is withheld for anonymity. The herbal products were imported to Norway under Norwegian Medicines Agency license no. 16/04551–2.

For DNA barcoding, nrITS reference sequences of 118 accessions belonging to 56 *Veronica* species were compiled into a barcode reference database, consisting partly of sequences mined from NCBI/GenBank and partly of sequences generated within the project from specimens collected in 2014 and 2015 (Supplementary Table S2). For the phytochemical analyses, aerial parts of *V. officinalis* and *V. chamaedrys* (samples marked with an asterisk in Supplementary Table S2) were used as references for the identification and quantification of the main compounds. Voucher specimens of the plant material used in this study are deposited in the Herbarium of the Alexandru Borza Botanical Garden (CL) of Babes-Bolyai University, Cluj-Napoca, Romania (Supplementary Table S2).

### Phytochemical Analysis

### High-Performance Liquid Chromatography-Mass Spectrometry (HPLC-MS)

Extracts were prepared from plant material and herbal products following established procedures (Crişan et al., 2010; Mocan et al., 2015a,b), with slight modifications. One gram of each sample was extracted with 10 ml of 70% ethanol (HPLC grade) at room temperature for 1 h in a sonication bath (Polsonic 3, Polsonic Inc., Poland). The extracts were filtered through MN 615 filter paper (Macherey-Nagel GmbH, Germany) and stored at 4°C until further analysis. Prior to HPLC-MS analysis the extracts were syringe filtered with a 0.45 μm nylon membrane (Whatman Inc., United States).

Target *Veronica* iridoids (aucubin, catalpol, catalposide, and veronicoside) from all sixteen herbal products as well as aerial plant parts were analyzed by HPLC-MS on a Agilent 1100 liquid chromatography system equipped with a binary pump, autosampler, thermostat and detector (all 1100 Series from Agilent Inc., United States). The system was controlled with Data Analysis software (version B01.03, Agilent Inc., United States). The separation was carried out on an Atlantis HILIC 3.5 μm (100 mm × 3.0 mm i.d.) (Waters Inc., United States) column equipped with an online 0.2 μm filter (Agilent Inc.), at a working temperature of 40°C, a flow rate of 0.75 ml/min and an injection volume of 6 μl. A binary gradient system with eluent (A) 0.1% acetic acid and 20 μM sodium acetate in water, and eluent (B) 0.1% acetic acid and 20 μM sodium acetate in acetonitrile was used for the analyzed samples with the following gradient: 95–80% B (1–5 min). The HPLC system was coupled with an Agilent Ion Trap 1100 SL mass spectrometer equipped with an electrospray ionisation (ESI) source and operated in the positive mode with a scan range between 360 and 680 m/z, to identify the target compounds based on their sodium adducts (M+23 m/z): aucubin (369 m/z), catalpol (385 m/z), catalposide (505 m/z) and veronicoside (489 m/z), and by comparison with analytical standards in the same chromatographic conditions. The capillary voltage was set to 4 kV and the capillary temperature to 325°C. Nitrogen (N_2_) was used as dry gas with a dry flow of 12 l/min and a pressure of 60 psi for the nebulizer. For quantitation of the iridoids, stock solutions of the four commercially available standards substances (Sigma–Aldrich, St. Louis, Missouri, United States) were prepared in acetonitrile, and different concentrations of each standard were used. All calibration curves yielded a coefficient of determination of *R*^2^ ≥ 0.99. The results are expressed as μg per g of dry weight material (μg/g dw).

### Genetic Analysis

#### DNA Extraction, PCR Amplification and DNA Sequencing

The total DNA was extracted from 300 mg of each homogenized herbal product or silica gel dried leaves, using a modified CTAB extraction protocol (Doyle and Doyle, 1987; Kool et al., 2012). Two extraction negative controls were included to screen for contamination and cross-contamination. Total DNA extracts of the herbal products were quantified and assessed for fragmentation with a Fragment Analyzer (Advanced Analytical Technologies, Inc., Ankeny, IA, United States) using the DNF-488-33 HS (High Sensitivity) genomic DNA Reagent Kit (50 – 40000 bp). Total DNA extracts of silica gel dried leaves were quantified with a Qubit 2.0 fluorometer (Life Technologies, Carlsbad, CA, United States) using the dsDNA BR (Broad-Range) kit. PCR amplification using nrITS primers, ITS-4 and ITS-5 (White et al., 1990), was done on purified total DNA from the dried leaves, using a final reaction volume of 25 μl, including 2.5 μl 10X reaction buffer II (supplied with the polymerase), 2.5 μl 25 mM MgCl_2_ (supplied with the polymerase), 0.2 μl 5 U/ml AmpliTaq DNA Polymerase (Applied Biosystems, Foster City, CA, United States), 2.5 μl 10 μM dNTPs (Applied Biosystems, Foster City, CA, United States), 2.5 μl 10 μM of each primer (Sigma–Aldrich, St. Louis, Mo, United States), 2.5 μl 1 mg/ml bovine serum albumen (BSA) (Roche Diagnostic GmbH, Mannheim, Germany), 9.3 μl of Milli-Q ultrapure water and 0.5 μl of template DNA solution (1 ng/μl). The PCR cycling protocol consisted of initial denaturation at 94°C for 2.5 min, followed by 32 cycles of denaturation at 94°C for 30 s, annealing at 53°C for 30 s, and elongation at 72°C for 50 s, followed by a final elongation step at 72°C for 4 min. Three PCR negative controls were included per amplification to control for external and cross-sample contamination. 6 μl of each PCR products was purified using 2 μl 10 times diluted ExoSAP-IT (USB Corporation, Cleveland, OH, United States) by incubation at 37°C for 45 min followed by 15 min at 80°C. Sequencing was performed using the ABI BigDye Terminator sequencing buffer and the v3.1 Cycle Sequencing kit (Applied Biosystems, Foster City, CA, United States) on an ABI3130XL automated sequencer (Applied Biosystems, Foster City, CA, United States). The sequences were edited, and the forward and reverse sequences assembled using SeqTrace (Stucky, 2012). The sequences were submitted to NCBI/GenBank and their accession numbers are listed in the Supplementary Table S2.

#### Barcode Gap Analysis

The reference sequences of 118 accessions belonging to 56 *Veronica* species were aligned using AliView (Larsson, 2014), and pairwise genetic distances between the accessions were calculated using MEGA 6.0 (Tamura et al., 2013) for each of the following regions: nrITS1, nrITS2, and the entire nrITS. Automatic Barcode Gap Discovery (ABGD) (Puillandre et al., 2012) was used to determine the genetic distance threshold and automatically define species delimitation hypotheses. ABGD determines the number of groups (hypothetical species) within a dataset using pairwise sequence distances and two input variables, prior intraspecific diversity (*P*) and minimum gap width (*X*) (Puillandre et al., 2012). Based on this information ABGD makes an initial partition of the supplied sequences into candidate species groups based on a statistically inferred barcode gap and then applies recursive partitions to each group to generate subsequent partitions into candidate species until no further splitting occurs (Puillandre et al., 2012). The minimum gap width (*X*) was set to *X* = 1.0, the prior intraspecific diversity (*P*) was set to range from 0.001 to 0.1, and the *p*-distance, Jukes-Cantor (JC) and Kimura 2 Parameter (K2P) distance metrics were used in the ABGD analyses (Jukes and Cantor, 1969; Kimura, 1980), resulting in 18 analyses (6 per dataset). Default values were used for all other parameters (steps: 10 and Nb bins: 20). The total numbers of groups corresponding to the prior intraspecific divergence (*P*) values, ranging from 0.001 to 0.1, were recorded (Supplementary Table S3).

To determine whether *V. officinalis* can be unambiguously differentiated from its main adulterant, *V. chamaedrys*, using molecular operational taxonomic units (MOTUs), the intra- and interspecific variation of all reference sequences of these species, respectively, 16 accessions belonging to *V. officinalis* and 9 to *V. chamaedrys*, were analyzed based on pairwise comparison of nrITS genetic distances using Taxon DNA/SpeciesIdentifier v 1.7.8 (Meier et al., 2006).

#### DNA Metabarcoding

Fusion primers of the internal transcribed spacers nrITS1 and nrITS2, based on primers 17SE and 5.8I1, and 5.8I2 and 26SE (Sun et al., 1994) (Biolegio B.V., the Netherlands), were used to generate PCR based amplicons. Forward primers were fused with a unique 10 bp multiplex identifier (MID) tags and the reverse primers with a truncated version of adapter P1 (trP1) tags. Expected amplicon sizes were 300–350 bp. PCR reactions on DNA extracted from the herbal products were carried out in final reaction volumes of 25 μl including 0.5 μl of template DNA solution (ranging from 0.5 to 2 ng/μl), 5 μl 5X Q5 reaction buffer (New England Biolabs Inc., United Kingdom), 1.5 μl 10 μM of each primer (Biolegio B.V., the Netherlands), 0.5 μl 10 mM dNTPs, 0.25 μl 20 U/μl Q5 High-Fidelity DNA Polymerase (New England Biolabs Inc., United Kingdom), 5 μl 5X Q5 High GC enhancer and 10.75 μl of Milli-Q ultrapure water. The PCR cycling protocol consisted of initial denaturation at 98°C for 30s, followed by 35 cycles of denaturation at 98°C for 10s, annealing at 56°C for nrITS1 or 71°C for nrITS2 for 30 s, and elongation at 72°C for 30 s, followed by a final elongation step at 72°C for 2 min. Eight PCR negative controls were included per amplification to control for external and cross-sample contamination.

Agencourt AMPure XP PCR purification (Beckman Coulter Inc., United States) was used for high-throughput purification of PCR amplicons. The size, purity and the molar concentration (nmol/l) of each amplicon library was measured using a Fragment Analyzer^TM^ (Advanced Analytical Technologies Inc., United States) with a DNF-910 dsDNA Reagent Kit (35–1,500 bp), equimolar pools (2 ng/μl/library) were prepared from the amplicon libraries using a Biomek 4000 Laboratory Automation Workstation (Beckman Coulter Inc., United States). To identify the optimum bead-to-template ratio for template preparation, the concentration of the purified pooled amplicon libraries was analyzed with a Fragment Analyzer^TM^ (Advanced Analytical Technologies, Inc., United States) using a DNF-488 High Sensitivity Genomic DNA Analysis Kit. The pooled Ion AmpliSeq libraries (Life Technologies, Thermo Fisher Scientific Inc., United States) for emulsion PCR and sequencing chip loading were prepared using an Ion Chef (LT). The DNA template concentration was adjusted to the number of Ion Sphere Particles (ISPs) and added to the emulsion PCR master mix. The template-positive ISPs were enriched and sequenced on an Ion Torrent Personal Genome Machine (LT) using Ion 318 v2 chips (LT) and the Ion PGM Sequencing 400 kit (LT). Sequencing read data was initially processed and demultiplexed based on MIDs into FASTQ files using Ion Torrent Suite Software version 5.0.4 (LT).

#### Bioinformatics Analysis

Sequencing reads were processed using the HTS barcode-checker pipeline freely available at https://github.com/naturalis/HTS-barcode-checker (Lammers et al., 2014). The nrITS1 and nrITS2 primer sequences were used to split the reads based on the primer sequence. Read lengths and Phred quality scores were assessed using PRINSEQ (Schmieder and Edwards, 2011), and based on these values reads with a mean Phred quality score of less than 26 and a length of less than 300 bp were filtered out. The remaining reads were trimmed to a maximum length of 350 bp. Reads were clustered into MOTUs using three different sequence similarity thresholds during clustering, respectively, 97, 99 and 100%, with a minimum of 10 reads per cluster using CD-HIT-EST (Li and Godzik, 2006). A representative sequence from each MOTU was taxonomically assigned using local Basic Local Alignment Search Tool (local BLAST) (Altschul et al., 1990), with a maximum e-value of 0.05 and a minimum hit length of 100 bp, against a reference database consisting of a local copy of the NCBI nucleotide database (Benson et al., 2013). Species level identifications were assigned when similarity to the reference barcode matched at >99%.

## Results and Discussion

### Phyotochemical Analysis

#### High-Performance Liquid Chromatography-Mass Spectrometry (HPLC-MS)

Iridoids and iridoid glycosides are specific chemotaxonomic markers for the genus *Veronica*, and most *Veronica* species contain the iridoid glycosides aucubin and/or catalpol as well as one or more 6-*O*-esters of catalpol (i.e., veronicoside, catalposide) (Afifi-Yazar and Sticher, 1980; Taskova et al., 2002, 2004; Jensen et al., 2005). The main known ethnopharmacological indication for *Veronica* species are inflammatory diseases (rheumatism, stomach ulcer) or respiratory ailments (Scarlat et al., 1985; Mocan et al., 2015a), and several studies have shown that iridoid glycosides in *Veronica* are linked to this therapeutic effects (Scarlat et al., 1985; Gründemann et al., 2013; Ignjatović, 2015). Although regulatory guidelines to establish quality control or authentication of *Veronica* herbal products are lacking, the presence and concentration of specific iridoid glycosides has been shown to enable authentication of *Veronica* herbal products (Crişan et al., 2010; Gründemann et al., 2013).

The HPLC-MS results from the present study show the characterization of *Veronica* herbal products against four commercially available iridoid glycosides, namely aucubin, catalpol, veronicoside and catalposide (**Table 2**). The two most commonly identified compounds in the examined herbal products and reference plant material were aucubin and catalpol. From a pharmaceutical point of view, the concentrations of the compounds present in different herbal remedies or plant materials cannot be neglected as they are directly linked to their pharmaceutical efficacy and effectiveness. Aucubin was found in fifteen herbal products samples as well as in the reference plant material of both species (*V. officinalis* and *V. chamaedrys*), with the highest concentration in sample 9. Aucubin concentrations in the herbal products and reference plant material show a large variation with concentrations ranging from 0.33 to 39.73 μg/g. Catalpol was found in 13 herbal products, with the highest concentration in sample 11, but was missing in two of the reference plant samples (*V. off* 4 and *V. cha* 1). No iridoid glycosides were identified in sample 14, a lozenge with nineteen ingredients listed on the label. Jensen et al. (2005) concludes that aucubin and/or catalpol is/are present in all *Veronica* species, implying that if sample 14 contains any material of *Veronica* this would be below the level of detection of the HPLC-MS platform we used in this study, and consequently unlikely to exert any pharmacological activity. Concerning the other two 6-*O*-esters of catalpol, veronicoside was present in five herbal products and in all *V. officinalis* reference material, with highest concentration in sample *V. off* 1, while catalposide was present in nine herbal products, with the highest concentration in sample 8, but none of the investigated reference plant materials contained catalposide. Jensen et al. (2005) states that although aucubin and/or catalpol are present in all *Veronica* species, *V. micrantha* is the only species in *Veronica* subgenus *Chamaedrys*, to which *V. chamaedrys* belongs, that contains aucubin. However, this assumption was later rejected by Crişan et al. (2010) that identified both aucubin and catalpol in *V. chamaedrys*, and as well as by the results of this study.

**Table 2:** Concentrations of the four main iridoid glycosides in the analyzed herbal products and the *Veronica* reference plant material (details about the herbal products can be found in Supplementary Table S1).

Product no.	Concentrations (in μg/g)			
	
	Veronicoside	Catalposide	Aucubin	Catalpol
1	n/c	0.48 ± 0.02	1.19 ± 0.03	0.28 ± 0.01
2	n/c	n/c	1.58 ± 0.02	1.05 ± 0.04
3	n/c	3.38 ± 0.11	13.77 ± 0.52	1.52 ± 0.04
4	0.24 ± 0.01	2.10 ± 0.08	29.17 ± 1.03	5.42 ± 0.18
5	n/c	n/c	0.33 ± 0.01	0.56 ± 0.02
6	n/c	n/c	20.79 ± 0.73	n/c
7	n/c	n/c	6.01 ± 0.13	n/c
8	n/c	4.56 ± 0.16	18.24 ± 0.71	5.82 ± 0.12
9	n/c	0.22 ± 0.01	39.73 ± 1.17	2.33 ± 0.09
10	n/c	n/c	26.88 ± 0.95	3.97 ± 0.13
11	8.81 ± 0.39	0.77 ± 0.02	11.78 ± 0.43	11.46 ± 0.37
12	9.06 ± 0.41	2.01 ± 0.08	7.31 ± 0.29	9.63 ± 0.38
13	2.74 ± 0.11	2.08 ± 0.07	4.16 ± 0.17	1.92 ± 0.08
14	n/c	n/c	n/c	n/c
15	n/c	n/c	0.64 ± 0.02	0.43 ± 0.01
16	1.01 ± 0.03	0.37 ± 0.01	1.11 ± 0.04	1.12 ± 0.02
*V. off* 1	36.07 ± 1.8	n/c	11.32 ± 0.55	10.04 ± 0.45
*V. off* 2	0.29 ± 0.01	n/c	1.94 ± 0.07	3.32 ± 0.15
*V. off* 3	1.72 ± 0.03	n/c	1.53 ± 0.06	0.48 ± 0.01
*V. off* 4	0.27 ± 0.01	n/c	0.41 ± 0.02	n/c
*V. cha* 1	n/c	n/c	3.39 ± 0.11	n/c
*V. cha* 2	n/c	n/c	6.12 ± 0.29	0.87 ± 0.03
*V. cha* 3	n/c	n/c	7.44 ± 0.32	0.93 ± 0.02
*V. cha* 4	n/c	n/c	7.52 ± 0.27	2.38 ± 0.10


### Genetic Analyses

#### Barcode Gap Analysis

Species-level identification using DNA barcoding and metabarcoding requires that the level of intraspecific genetic divergence is lower than the interspecific genetic divergence (Meyer and Paulay, 2005), and several identification approaches are based on setting a cut-off threshold above which a query sequence is considered to be distinct from a reference sequence. Defining an optimal threshold is a controversial debate (Hebert et al., 2004; Meyer and Paulay, 2005; Smith et al., 2005; Wiemers and Fiedler, 2007; Chen et al., 2013). Previous studies proposed different thresholds of genetic distances, for instance, 1% (Ratnasingham and Hebert, 2013), 3% (Smith et al., 2005) or the 10X rule where the interspecific distances are considered to be 10 times greater than intraspecific distances (Hebert et al., 2004), but each has shortcomings for general application (Meier et al., 2008; Rach et al., 2008; Bertolazzi et al., 2009).

In this study we used ABGD to assess the ‘barcode gap’ for the *Veronica* genus, with the specific aim of evaluating if nrITS has sufficient discriminatory power to differentiate *V. officinalis* from its main adulterant, *V. chamaedrys*. The three distance metrics used for the analyses produced identical initial partitions within each dataset and for all the values of prior intraspecific diversity (*P*), but with a slight difference between the datasets, respectively, 14 groups for nrITS and nrITS2 and 13 for nrITS1. The recursive partitions resulted in a variable number of groups within each dataset depending on *P*, respectively, with 18, 22, 37, and 53 groups for nrITS dataset, 14, 19, and 37 groups for nrITS1, and 16, 23, 31, and 54 groups for nrITS2 data set.

Infra- and interspecific divergence based on pairwise comparison of nrITS genetic distances of *V. officinalis* and *V. chamaedrys* (Supplementary Table S4) helped to identify an optimal clustering threshold for analysis of high-throughput sequence data and accurate molecular identification and discrimination of the two species. Pairwise intraspecific genetic distances did not exceed 1.5%, and furthermore 93.8% of the pairwise intraspecific distances were below 1%. Pairwise interspecific genetic distances, on the other hand, ranged from 18 to 22%. The pairwise comparisons showed that applying 97% or more similarity as a clustering threshold will confidently discriminate *V. officinalis* and *V. chamaedrys.* These results are consistent with the ABGD analysis in which the sequences belonging to *V. officinalis* and *V. chamaedrys* were assigned to well defined independent groups using an *a priori* defined genetic threshold of 1%.

#### DNA Metabarcoding

The quantity of the total DNA extracted from the sixteen herbal products varied between samples. Total DNA concentration measurement gave detectable results for 15 of the samples (93.7%), with DNA concentrations ranging from 0.06 to 87.32 ng/μl, and nrITS1 and nrITS2 amplicons were obtained for all these samples. One sample, marketed as a lozenge, did not yield a measurable DNA concentration and no amplicons of nrITS1 and nrITS2 were obtained from this sample (Supplementary Table S5).

The raw data before demultiplexing consisted of 2,638,101 sequences, with an average of 82,440 sequences per sample for each marker. Sequencing was successful for thirteen herbal products (81.3%) for both nrITS1 and nrITS2 and they were used for further analysis (**Table 3**). Three products, two herbal teas (5, 16) and the lozenge (14), did not yield reads or MOTUs after applying the quality filtering criteria and were excluded from further analyses. A total of 63,929 sequences passed our trimming and filtering quality criteria (2.4% of reads), including 19,788 nrITS1 reads and 44,141 nrITS2 reads (**Supplementary Table S6**). The metabarcoding data is used for qualitative evaluation only, to determine presence of identified taxa, and not for quantitative assessment of relative species abundance based on read numbers, as too many variables impact the values, such as the availability of DNA that can be removed or degraded during the harvesting, drying, storage, transportation and processing (Bombardelli, 1991; Novak et al., 2007), as well as variation in nrITS copy numbers, primer annealing and PCR amplification biases (Pawluczyk et al., 2015). Furthermore, incomplete reference databases and incorrectly identified sequences in GenBank can impact taxonomic assignments.

**Table 3:** Overview of the results of three clustering thresholds (97, 99, and 100%).

Sample #	nrITS1+nrITS2 # reads before demultiplexing	nrITS21 # reads	nrITS2 # reads	97%		99%		100%	
						
				# MOTUs	# Species	# MOTUs	# Species	# MOTUs	# Species
1	163443	80255	78683	15	6	75	11	108	12
2	180106	104096	67500	17	11	71	17	79	16
3	158877	85618	69168	19	7	53	13	188	12
4	440956	299997	114335	47	12	142	23	191	16
5^∗^	34	8	18	0	0	0	0	0	0
6	217323	131223	74700	16	7	27	7	47	7
7	400607	174347	192110	49	25	99	32	191	26
8	309978	73352	213401	30	16	107	23	303	19
9	80179	58175	16627	25	14	36	13	49	12
10	62198	45321	15903	12	5	47	7	49	4
11	254965	186423	47991	33	14	64	16	77	10
12	122393	63939	55331	14	7	33	5	108	4
13	26001	16464	7830	0	0	4	2	12	2
14^∗^	19	10	6	0	0	0	0	0	0
15	220984	70947	131918	10	4	66	4	170	4
16^∗^	38	12	18	0	0	0	0	0	0
**Total**	**2638101**	**1390187**	**1085539**	**287**	**91**	**824**	**109**	**1572**	**80**


*^∗^Samples excluded from subsequent data analysis.*

The choice of similarity clustering thresholds (>97, >99, and 100%) impacted the number and size of assigned MOTUs (**Table 3**). Setting the clustering threshold to 100% yielded 1572 MOTUs, 99% yielded 824 MOTUs, and 97% yielded only 287 MOTUs. The low number of MOTUs obtained using the 97% clustering threshold is likely the result of multiple species being grouped together into the same MOTU as 3% divergence far exceeds interspecific variation in many genera (Yang and Rannala, 2017). Setting the clustering threshold to 100%, on the other hand, likely resulted in more than one MOTU being formed for a single taxonomic species as a result of infraspecific variation and/or sequencing errors between reads. Here we followed previous studies in using a 99% clustering threshold (Ghorbani et al., 2016; Raclariu et al., 2017; Veldman et al., 2017). Furthermore, to limit the impact of sequencing errors, which are known to affect the Ion Torrent sequencing platform (Loman et al., 2012; Salipante et al., 2014) and which could lead to the formation of false MOTUs, we used only the clusters that contained a minimum 10 reads.

**FIGURE 1:**
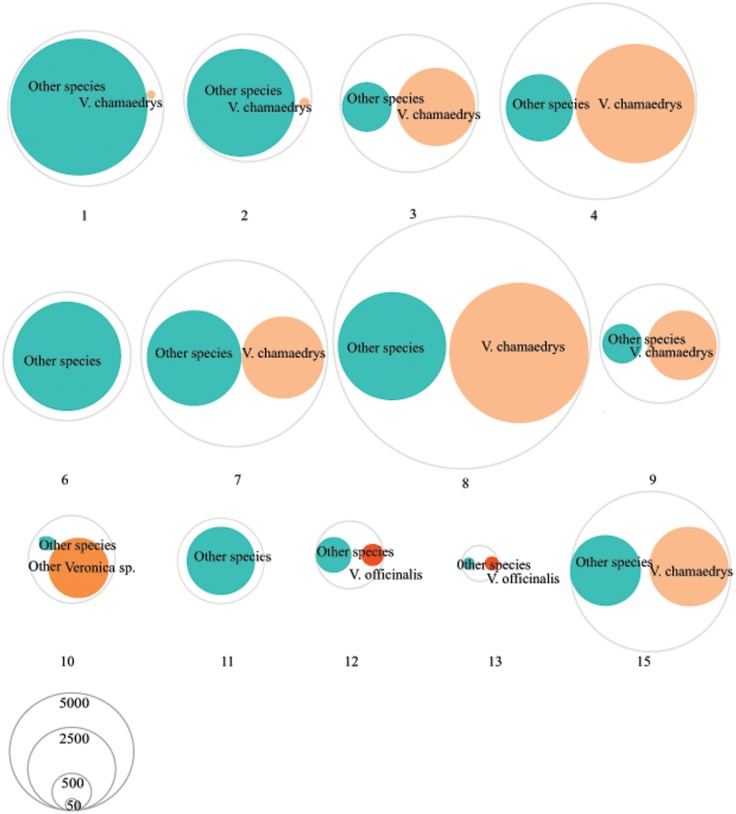
*Veronica* and other species detected in the herbal products using DNA metabarcoding. Each numbered circle represents a product (more information about these products can be found in Supplementary Table S1). The size of circles corresponds to the relative abundance of the reads.

The 824 MOTUs that were retained using a 99% similarity clustering threshold were further identified using BLAST as 109 different species (**Table 3** and **Supplementary Table S6**). Based on only nrITS1 we detected a total of 64 different species and on only nrITS2 61 species (**Supplementary Table S6**). Based on both markers, the number of species detected per sample ranged from 2 to 32, with an average of 8 species per sample. Only two of the seven (28.6%) single ingredient samples (those listing *V. officinalis* as the only ingredient) contained *V. officinalis*, three others contained *V. chamaedrys* and one contained other *Veronica* species, and all products contained additional species, ranging from 1 to 21 species per product. None of the nine retained multiple ingredient samples (i.e., those listing *V. officinalis* and other species on the label) contained only the listed ingredients, and none contained *V. officinalis* (**Figure 1**). The overall ingredient fidelity (detected species from product label/total number of species on label) was 9.5% for the multiple ingredient products, and 17% for all products. The target species *V. officinalis* was detected in only 2 products (15%), whereas *V. chamaedrys* was detected in eight (62%) of the retained samples. These results are supported by the pairwise comparison of the genetic distances that showed that *V. officinalis* could be unambiguously differentiated from *V. chamaedrys* using standard arbitrary clustering thresholds (Supplementary Table S4). In sample 10 a mixture of several *Veronica* species was detected, including *V. bombycina* Boiss. & Kotschy, *V. filiformis* Sm., *V. pectinata*, *V. tenuifolia* Asso, *V. krumovii* (Peev) Peev and *V. chamaedrys*. The presence of these species can be explained as substitutes, admixtures or contaminants. It should be noted that the general monograph number 1433 on ‘Herbal drugs’ of the European Pharmacopoeia allows up to 2% foreign matter, unless this is differently indicated in a specific herb monograph (EDQM - Council of Europe, 2014). Presence of these other *Veronica* species might not be in breach of the European Pharmacopoeia but it does indicate a lapse in the value chain of this product. The same applies to the 62% products that contained *V. chamaedrys* instead of *V. officinalis*.

## Conclusion

This study used HPLC-MS and DNA metabarcoding to authenticate and detect adulteration or admixture of *V. officinalis* with *V. chamaedrys* in *Veronica* herbal products. HPLC-MS is useful for qualitative and quantitative analysis of constituents in medicinal plants and herbal products and its use has increased in recent years (Steinmann and Ganzera, 2011; Bansal et al., 2014), but constraints are the expensive chemical reference standards (some of which are still unavailable for biologically active target components), sensitivity to the type of raw material and manufacturing process, and lack of resolution in distinguishing plant species (Bansal et al., 2014). Our HPLC-MS results show that distinction of *V. officinalis* from other *Veronica* species based on the targeted iridoid glycosides, aucubin, catalpol, veronicoside, and catalposide is difficult in pure products, and impossible in multiple ingredient products. General application of HPLC-MS to detect substitution and/or adulteration of highly processed and multi-ingredient herbal products is challenging, and this is corroborated by our findings.

Several authors have advocated the use of DNA barcoding and metabarcoding in herbal product authentication and pharmacovigilance (Coghlan et al., 2012; Newmaster et al., 2013; Cheng et al., 2014; De Boer et al., 2015; Palhares et al., 2015; Ivanova et al., 2016), due to its cost effectiveness and ability to disclose plant species diversity within products. Our DNA metabarcoding results corroborate previous research that used metabarcoding to authenticate herbal products (Coghlan et al., 2012, 2015; Cheng et al., 2014; Ivanova et al., 2016; Veldman et al., 2017). In this study we confirm its applicability to test for presence of *V. officinalis* and simultaneously to detect substitution, adulteration and/or admixture of other *Veronica* species. Using DNA metabarcoding we found that only 15% of the products contained *V. officinalis*, whereas 62% of the products contained *V. chamaedrys*. Furthermore, we found that all the investigated herbal products contained other species not listed on the label. Limitations of DNA metabarcoding are its susceptibility to various factors, such as quality, processing state and product type of the extracted material, as well as several variables related to the methodological framework, such as DNA extraction procedures, primers, markers, amplification protocols for the library preparation sequencing platform, filtering, quality thresholds and chimera removal, and clustering thresholds.

Despite limitations of the methodology, our DNA metabarcoding results show that there is a need to study herbal products derived from traditional medicine, and to increase consumer confidence by advocating and promoting a higher standard of quality in herbal products. In recent years, the scientific community has been showing an increasing interest in finding adequate and comprehensive methodologies to investigate such complex products, focusing on their entire value chain, from the raw material to the product on the shelf.

## Supporting Information

Ion-Torrent amplicon read data is deposited in DRYAD doi: 10.5061/dryad.606ks

## Author Contributions

MI, HB, GC, and AR conceived the experiment. AR collected the material and carried out the molecular lab work and analysis. MP assisted with the molecular lab work. AM, LV, and GC carried out the phytochemical lab work and analyses. AR wrote the manuscript together with HB and AB. All authors contributed to and approved the final version of the manuscript.

## Conflict of Interest Statement

The authors declare that the research was conducted in the absence of any commercial or financial relationships that could be construed as a potential conflict of interest.

## Supplementary Material

**TABLE S6 |** *Veronica* HTS reads and identified MOTUs per product at 99% threshold.
