# Erratum: *Veronica officinalis* Product Authentication Using DNA Metabarcoding and HPLC-MS Reveals Widespread Adulteration with *Veronica chamaedrys*

**DOI:** 10.3389/fphar.2017.00957

**Published:** 2017-12-19

**Authors:** Frontiers Production Office

**Affiliations:** Frontiers Production Office, Frontiers, Lausanne, Switzerland

**Keywords:** adulteration, DNA metabarcoding, herbal products, HPLC-MS, *Veronica chamaedrys*, *Veronica officinalis*

Due to a typesetting error, affiliation 5 was incorrectly identified as “Centre for Ecological and Evolutionary Synthesis (CEES), Department of Biosciences, University of Oslo, Oslo, Norway,” the correct affiliation is: “Department of Pharmaceutical Technology and Biopharmaceutics, Iuliu Hatieganu University of Medicine and Pharmacy, Cluj-Napoca, Romania.” Also, affiliation 6 was missing “University of Oslo,” the correct affiliation 6 is: “Department of Biosciences, Centre for Ecological and Evolutionary Synthesis (CEES), University of Oslo, Oslo, Norway.”

The publisher apologizes for this error and the original article has been updated to reflect this. This error does not change the scientific conclusions of the article in any way.

