# Supplementary material for: Veronica officinalis Product Authentication Using DNA Metabarcoding and HPLC-MS Reveals Widespread Adulteration with Veronica chamaedrys
Source: Front Pharmacol. 2017 Jun 19;8:378. doi: 10.3389/fphar.2017.00378 (PMC5474480; doi:10.3389/fphar.2017.00378)
Supplement: Supplementary file 2 [file Table_1.PDF]

**Supplementary Table S1.** Information about herbal products

| Sample no. | Species listed on label | Product type | Country of origin | Country of acquisition | Vendor type | Product clasification | Scientific names of the plant ingredients |
|---|---|---|---|---|---|---|---|
| 1 | 7 | Extract | Romania | Romania | Pharmacy | Food supplement | *Calendula officinalis* L. |
| | | | | | | | *Cichorium intybus* L. |
| | | | | | | | *Agrimonia eupatoria* L. |
| | | | | | | | *Centaurium erythraea* Rafn. (syn. *Centaurium umbellatum* Gilib) |
| | | | | | | | *Veronica officinalis* L. |
| | | | | | | | *Frangula alnus* Mill. (syn. *Rhamnus frangula* L.) |
| | | | | | | | *Foeniculum vulgare* Mill. |
| 2 | 7 | Extract | Romania | Romania | Pharmacy | Food Supplement | *Calendula officinalis* L. |
| | | | | | | | *Achillea millefolium* L. |
| | | | | | | | *Melissa officinalis* L. |
| | | | | | | | *Hypericum perforatum* L. |
| | | | | | | | *Veronica officinalis* L. |
| | | | | | | | *Gentiana asclepiadea* L. |
| | | | | | | | *Silybum marianum* (L.) Gaertn. |
| 3 | 1 | Capsules | Romania | Romania | E-commerce | Food Supplement | *Veronica officinalis* L. |
| 4 | 1 | Herbal tea | Romania | Romania | E-commerce | Food Supplement | *Veronica officinalis* L. |
| 5 | 9 | Herbal tea | Romania | Romania | Herbal shop | Food Supplement | *Galium aparine* L. |
| | | | | | | | *Arctium lappa* L. |
| | | | | | | | *Achillea millefolium* L. |
| | | | | | | | *Taraxacum campylodes* G.E.Haglund (syn. *Taraxacum officinale* (L.) Weber ex F.H.Wigg.) |
| | | | | | | | *Galium verum* L. |
| | | | | | | | *Veronica officinalis* L. |
| | | | | | | | *Juglans regia* L. |
| | | | | | | | *Stellaria media* (L.) Vill. |
| | | | | | | | *Fumaria officinalis* L. |
| 6 | 1 | Herbal tea | Unknown | Romania | Market | Food Supplement | *Veronica officinalis* L. |
| 7 | 9 | Herbal tea | Romania | Romania | Herbal shop | Food Supplement | *Carum carvi* L. |
| | | | | | | | *Coriandrum sativum* L. |
| | | | | | | | *Mentha × piperita* L. |
| | | | | | | | *Matricaria chamomilla* L. |
| | | | | | | | *Veronica officinalis* L. |
| | | | | | | | *Taraxacum campylodes* G.E.Haglund (syn. *Taraxacum officinale* (L.) Weber ex F.H.Wigg.) |
| | | | | | | | *Cichorium intybus* L. |
| | | | | | | | *Stevia rebaudiana* (Bertoni) Bertoni |
| | | | | | | | *Zingiber officinale* Roscoe |
| 8 | 3 | Herbal tea | Romania | Romania | Herbal shop | Food Supplement | *Cynara scolymus* L. |
| | | | | | | | *Veronica officinalis* L. |
| | | | | | | | *Equisetum arvense* L. |
| 9 | 1 | Herbal tea | Romania | Romania | Herbal shop | Food Supplement | *Veronica officinalis* L. |
| 10 | 1 | Herbal tea | Unknown | Romania | Market | Unknown | *Veronica officinalis* L. |
| 11 | 5* | Herbal tea | Romania | Romania | Supermarket | Food Supplement | *Veronica officinalis* L. |
| | | | | | | | *Fraxinus excelsior* L. |
| | | | | | | | *Salix* sp. |
| | | | | | | | *Betula pendula* Roth |
| | | | | | | | *Juniperus communis* L. |
| 12 | 1 | Herbal tea | Poland | Poland | Herbal shop | Food Supplement | *Veronica officinalis* L. |
| 13 | 1 | Herbal tea | Unknown | Austria | Pharmacy | Unknown | *Veronica officinalis* L. |
| 14 | 19 | Candy | Austria | Austria | Pharmacy | Unknown | *Achillea millefolium* L. |
| | | | | | | | *Glycyrrhiza glabra* L. |
| | | | | | | | *Foeniculum vulgare* Mill. |
| | | | | | | | *Sanguisorba officinalis* L. |
| | | | | | | | *Althaea officinalis* L. |
| | | | | | | | *Plantago lanceolata* L. |
| | | | | | | | *Sambucus* sp. |
| | | | | | | | *Thymus serpyllum* L. |
| | | | | | | | *Salvia officinalis* L. |
| | | | | | | | *Mentha × piperita* L. |
| | | | | | | | *Rubus vestitus* Weihe (syn. *Rubus fruticosus* G.N.Jones) |
| | | | | | | | *Matricaria chamomilla* L. |
| | | | | | | | *Pimpinella anisum* L. |
| | | | | | | | *Centaurea cyanus* L. |
| | | | | | | | *Primula veris* L. |
| | | | | | | | *Tagetes erecta* L. |
| | | | | | | | *Veronica officinalis* L. |
| | | | | | | | *Althaea officinalis* L. |
| | | | | | | | *Illicium verum* Hook.f. |
| 15 | 8 | Herbal tea | Romania | Romania | Pharmacy | Food supplement | *Silybum marianum* (L.) Gaertn. |

| | | | | | | | |
|---|---|---|---|---|---|---|---|
| | | | | | | | *Calendula officinalis* L. |
| | | | | | | | *Veronica officinalis* L. |
| | | | | | | | *Melissa officinalis* L. |
| | | | | | | | *Hypericum perforatum* L. |
| | | | | | | | *Achillea millefolium* L. |
| | | | | | | | *Gentiana asclepiadea* L. |
| | | | | | | | *Stevia rebaudiana* (Bertoni) Bertoni |
| 16 | 11 | Herbal tea | Poland | Poland | Herbal shop | Herbal medicine | *Galium verum* L. |
| | | | | | | | *Veronica officinalis* L. |
| | | | | | | | *Cirsium oleraceum* (L.) Scop. |
| | | | | | | | *Avena sativa* L. |
| | | | | | | | *Verbena officinalis* L. |
| | | | | | | | *Stachys officinalis* (L.) Trevis. |
| | | | | | | | *Citrus limon* (L.) Osbeck |
| | | | | | | | *Elsholtzia* sp. |
| | | | | | | | *Hypericum perforatum* L. |
| | | | | | | | *Heracleum mantegazzianum* Sommier & Levier |
| | | | | | | | *Humulus lupulus* L. |

*The label of product 11 mentions a mixture of 17 plant ingredients, but only 5 plant species are listed.
