# Supplementary material for: Veronica officinalis Product Authentication Using DNA Metabarcoding and HPLC-MS Reveals Widespread Adulteration with Veronica chamaedrys
Source: Front Pharmacol. 2017 Jun 19;8:378. doi: 10.3389/fphar.2017.00378 (PMC5474480; doi:10.3389/fphar.2017.00378)
Supplement: Supplementary file 3 [file Table_2.PDF]

Supplementary Table S2. Details of *Veronica* species included in this study: voucher specimens, geografic origin and NCBI/GenBank accessions. The nrITS sequences generated for the current study are highlighted in bold.

Taxon name	Sequence #	nrITS (GenBank accession)	Voucher ID (Collector, No., Herbarium)	Geografical origin of material
Veronica acinifolia L.	1	AF509798	M. Fischer s.n., WU	Greece: Lefkas
Veronica agrestis L.	1	AF509784	Albach 386, WU	Greece: Taygetos
Veronica allionii Vill.	1	AF509809	Kew 1993-1463, K	Italy: Cult.RBG Kew
Veronica alpina L.	1	AF509810	Benson 1998.08, UPS	Sweden: Abisko NR
	2	AF313013	Albach 184, WU	France
Veronica anagallis-aquatica L.	1	KJ630576	Safer s.n. 5.5.2007, FM04, OLD	Egypt
	2	KJ630575	Duman 2350, GAZI	Turkey
	**3**	**KY853235**	**Toth AR68RO, CJ**	**Romania: Gheorgheni, Harghita**
Veronica anagalloides Guss.	1	KJ829450	Albach 280, WU	Turkey
	2	KJ829449	07/042, DA	Czech Republic
Veronica aragonensis Stroh	1	KT361668	93528 (MO885-1), SALA	Spain: Granada, Huéscar, Sierra de la Sagra
	2	KT361667	121537 (AA14-6), SALA	Spain: Huesca, collado de Ceresa, Peña Montañesa
Veronica arvensis L.	1	KT361715	149232 (BR229-1), SALA	Spain
	2	DQ227328	M. Sheahan 9, K	-
	**3**	**KY853243**	**Toth AR89RO, CJ**	**Romania: Piatra Neamt, Neamt**
Veronica austriaca L.	1	KJ630593	Rojas-Andr s Martnez-Ortega & Girldez BR178-2, SALA	Austria
	2	AF313000	Albach70, BONN	Germany: Cult. BG Bonn
	**3**	**KY853239**	**Raclariu AR77NO, CJ**	**Norway: BG Oslo**
Veronica aznavourii Dörfl.	1	EU224205	G. Ertem 21471, ISTE	Turkey
Veronica bachofenii Heuff.	1	KJ425739	Martinez Ortega 908, SALA	Romania
Veronica baumgartenii Roem. & Schult.	1	AY144464	-	-
Veronica beccabunga L.	1	KJ630580	Toth s.n. (AR43), OLD	Romania
	2	AF313015	Albach 122, K	
	**3**	**KY853228**	**Popescu AR55RO, CJ**	**Romania: Turda Gorges, Cluj**
	**4**	**KY853229**	**Raclariu AR56RO, CJ**	**Romania: Gheorghieni, Harghita**
	**5**	**KY853230**	**Toth AR57RO, CJ**	**Romania: Gheorghieni, Harghita**
	**6**	**KY853231**	**Toth AR58RO, CJ**	**Romania: Gheorghieni, Harghita**
Veronica bellidioides L.	1	AF313010	Cult. Kew 1998-761, K	UK: Cult. Kew
Veronica catenata Pennell	1	KJ829473	Mastroguiseppe & Marsden 22, WS	USA
	2	KJ829472	07/032, DA	Czech Republic
Veronica caucasica M.Bieb.	1	AF486357	Albach 326, WU	Kazbegi, Georgia
Veronica chamaedrys L.	1	KJ630587	Toth s.n. (AR40), OLD	Romania
	2	DQ227329	M. Fay 149, K	-
	**3**	**KY853223**	**Raclariu AR49RO, CJ**	**Romania: Piatra Neamt, Neamt**
	**4**	**KY853224**	**Raclariu AR50RO, CJ**	**Romania: Cuiejdi, Neamt**
	**5**	**KY853225**	**Toth AR51RO, CJ**	**Romania: Harghita**
	**6***	**KY853226**	**Raclariu AR53RO, CJ**	**Romania: Secu Vaduri, Neamt**
	**7**	**KY853227**	**Raclariu AR54RO, CJ**	**Romania: Ceahlau, Neamt**
	**8**	**KY853234**	**Toth AR67RO, CJ**	**Romania: Gheorghieni, Harghita**
	**9**	**KY853238**	**Raclariu AR73RO, CJ**	**Romania: Izvoare, Neamt**

	10*	-	Toth AR87RO, CJ	Romania: Şumulău Ciuc, Harghita
	11*	-	Toth AR871RO, CJ	Romania: Izvorul Mureşului, Hargita
	12*	-	AR531RO, CJ	Romania: Nechit, Neamt
Veronica chamaepithyoides Lam.	1	AF509796	Raclariu UA 174, SALA	-
Veronica cymbalaria Bod.	1	AY850104	Fischer 85-16-28, WU	Greece: Corfu
	2	AY850103	Albach 251, WU	Turkey: Perge
Veronica erinoides Boiss. & Spruner	1	EU282103	M von Sternburg 25.7.2006, WU	Greece
	2	EU282102	M. von Sternburg 018, WU	Greece
Veronica filiformis Sm.	1	GU143559	Jossberger s.n., STU	Germany
Veronica fruticans Jacq.	1	EU282108	Schoenswetter & Frajman 3.8.2006, WU	Spain: Cataluña, Aiguestortes
	2	EU282107	Schoenswetter 7.7.2006, WU	Austria: Carinthia, Hohe
Veronica fruticulosa L.	1	KJ630615	OLD	Germany: Cult.
	2	AF313004	Albach 71, BONN	Germany: Cult. BG Bonn
Veronica gentianoides Vahl	1	AF313018	Albach 72, BONN	Germany: Cult. BG Bonn
Veronica glauca Sibth. & Sm.	1	AF313006	Chase s.n., K	UK: Cult. Kew1973-14554
Veronica hederifolia L.	**1**	**KY853242**	**Toth AR84RO, CJ**	**Romania: Piatra Neamt, Neamt**
Veronica kindlii Adamović	1	KJ630609	Kamelin et al. 29.6.1996, ALTB	Russia
Veronica longifolia L.	1	KJ630609	Kamelin et al. 29.6.1996, ALTB	Russia
	2	KJ630608	OLD	Germany: Cult
Veronica mampodrensis Losa & P.Monts.	1	DQ227331	Martinez Ortega 713, SALA	-
Veronica micrantha Hoffmanns. & Link	1	DQ227330	Martinez Ortega 1754, SALA	-
Veronica montana L.	1	AF313014	Albach151,WU	Germany: Oberkassel
Veronica multifida L.	1	KT361711	Albach 1143, OLD	Turkey
	2	KJ630599	Albach 1143, WU	Turkey
Veronica nummularia Gouan	1	DQ227335	Martinez Ortega 718, SALA	-
Veronica officinalis L.	1	AF313012	Albach & Chase 114, K (donated by S.Joseph)	UK
	2	DQ534900		Turkey: Arhavi, Artvin
	**3**	**KY853212**	**Raclariu AR25RO, CJ**	**Romania: Cuiejdi, Neamt**
	**4**	**KY853213**	**Raclariu AR26RO, CJ**	**Romania: Pangarati, Neamt**
	**5***	**KY853214**	**Toth AR27RO, CJ**	**Romania: Izvorul Muresului, Harghita**
	**6**	**KY853215**	**Raclariu AR28RO, CJ**	**Romania: Cozla, Piatra Neamt**
	**7***	**KY853216**	**Raclariu AR37RO, CJ**	**Romania: Ceahlau National Park, Neamt**
	**8**	**KY853217**	**Toth AR38RO, CJ**	**Romania: Orotva, Harghita**
	**9**	**KY853218**	**Toth AR42RO, CJ**	**Romania: Gheorghieni, Harghita**
	**10***	**KY853219**	**Toth AR43RO, CJ**	**Romania: Borzont, Harghita**
	**11**	**KY853220**	**Raclariu AR45RO, CJ**	**Romania: Secu Vaduri, Neamt**
	**12**	**KY853221**	**Toth AR46RO, CJ**	**Romania: Gheorghieni, Harghita**
	**13**	**KY853222**	**Toth AR47RO, CJ**	**Romania: Gheorghieni, Harghita**
	**14**	**KY853237**	**Raclariu AR72RO, CJ**	**Romania: Cuiejdi, Neamt**
	**15**	**KY853240**	**Kolar AR80PL, CJ**	**Poland: Chodziez**
	**16**	**KY853241**	**Kolar AR81SE, CJ**	**Sweeden: Vaxjo**
	17*	-	Raclariu AR371RO, CJ	Romania: Ceahlau National Park, Neamt
Veronica opaca Fr.	1	AY673617	Albach 332, WU	-

Veronica orientalis Mill.	1	AY741515	Albach 701, WU	Turkey
Veronica panormitana Tineo ex Guss.	1	KJ646889	Albach 1233, OLD	Turkey
	2	AY850100	Albach 402, WU	-
Veronica pectinata L.	1	AY144460	Struwe 1410, WU	-
	2	KT361712	688478 (CA6210-1), MA	Turkey
Veronica peduncularis M.Bieb.	1	KT361714	110319 (MO1554-3), SALA	Georgia
Veronica peregrina L.	1	KF724918	2012SH003	-
	2	AF313016	Lesica 5668, K	-
Veronica persica Poir.	1	KJ630604	Albach 979, MJG	Germany
	2	AF509785	Fay 175, K	UK
Veronica polita Fr.	1	KJ630605	AR19, OLD	Romania
	2	KT361716	149255 (Sanchez-Agudo s.n.), SALA	Spain
Veronica praecox All.	1	EU224206	Albach 855, MZJG	Austria
Veronica prostrata L.	1	KT361691	149317 (MS1239-3), SALA	Bulgaria
	2	KT361690	149312 (BR215-1), SALA	Switzerland
Veronica rosea Desf.	1	KT361693	149323 (DP783-2), SALA	Marocco
	2	KT361694	149324 (MO5502-5), SALA	Algeria
Veronica saturejoides Vis.	1	KJ630617	Surina 19.7.2006, WU	Bosnia and Herzegovina
	2	EU282101	Surina 5.7.2006, WU	-
Veronica scutellata L.	1	AF509805	Dobes 7026, WU	Austria
Veronica serpyllifolia L.	1	AY036879	-	-
	2	AF313017	Albach 64, WU	Germany: Waldstrasse, Bonn
	**3**	**KY853236**	**Raclariu AR71RO, CJ**	**Romania: Ceahlau National Park, Neamt**
	**4**	**KY853244**	**Toth AR90RO, CJ**	**Romania: Piatra Neamt, Neamt**
Veronica spicata L.	1	AY673622	Fay-Avon Gorge, K	-
	2	AY673621	371, UK	-
	**3**	**KY853233**	**Toth AR62RO, CJ**	**Romania: Sumulau, Harghita**
	**4**	**KY853245**	**Raclariu AR91RO, CJ**	**Norway: BG Oslo**
Veronica tenuifolia Asso	1	AY741516	Martinez-Ortega s.n. (D), SALA	-
	2	AF312996	Sandwith 4969, K	Spain: Sierra de Gidar
Veronica thessalica Benth.	1	KJ630618	Raus & Rogl 5072, SALA	Greece
	2	EU282106	Schoenswetter & Frajman 15.8.2006, WU	-
Veronica thymifolia Sm.	1	FJ848065	isolate="D Albach 832"	-
	2	KJ630592	Albach S434, OLD	Russia
Veronica triphyllos L.	1	KT361708	149334 (MS1247-1), SALA	Bulgaria
	2	KT361707	149333 (BR45-3), SALA	Turkey
Veronica turrilliana Stoj. & Stef.	1	AF313011	Albach 73, BONN	Germany: Cult. BG Bonn
Veronica urticifolia Jacq.	1	AF313011	Albach 73, BONN	Germany: Cult. BG Bonn
	**2**	**KY853232**	**Raclariu AR60RO, CJ**	**Romania: Ceahlau National Park, Neamt**
Veronica verna L.	1	AF509789	Albach 149, WU	Germany: Bad Kreuznach

*Specimens used for the HPLC-MS analyses
