# Supplementary material for: Veronica officinalis Product Authentication Using DNA Metabarcoding and HPLC-MS Reveals Widespread Adulteration with Veronica chamaedrys
Source: Front Pharmacol. 2017 Jun 19;8:378. doi: 10.3389/fphar.2017.00378 (PMC5474480; doi:10.3389/fphar.2017.00378)
Supplement: Supplementary file 4 [file Table_3.PDF]

Supplementary Table S3. Results of partitions generated by the Automatic Barcode Gap Discovery (ABGD)

	**Number of groups of the nrITS data set**						**Number of groups of the nrITS 1 data set**						**Number of groups of the nrITS2 data set**					
	**Initial partition**			**Recursive partition**			**Initial partition**			**Recursive partition**			**Initial partition**			**Recursive partition**		
	**X=1.0**						**X=1.0**						**X=1.0**					
**Prior intraspecific divergence (*P*)**	**JC**	**K2P**	**p**	**JC**	**K2P**	**p**	**JC**	**K2P**	**p**	**JC**	**K2P**	**p**	**JC**	**K2P**	**p**	**JC**	**K2P**	**p**
0.0010	14	14	14	53	53	53	13	13	13	37	37	37	14	14	14	55	54	54
0.0017	14	14	14	53	53	53	13	13	13	37	37	37	14	14	14	54	54	54
0.0028	14	14	14	37	37	37	13	13	13	37	37	37	14	14	14	54	54	54
0.0046	14	14	14	37	37	37	13	13	13	37	37	37	14	14	14	31	31	31
0.0077	14	14	14	22	22	22	13	13	13	19	19	19	14	14	14	23	23	23
0.0129	14	14	14	18	18	18	13	13	13	19	19	19	14	14	14	16	16	16
0.0215							13	13	13	14	14	14						

X=relative gap width; p= p-distance substitution model; JC=Jukes-Cantor substitution model; K2P=Kimura 2-parameters substitution model
