# Supplementary material for: Veronica officinalis Product Authentication Using DNA Metabarcoding and HPLC-MS Reveals Widespread Adulteration with Veronica chamaedrys
Source: Front Pharmacol. 2017 Jun 19;8:378. doi: 10.3389/fphar.2017.00378 (PMC5474480; doi:10.3389/fphar.2017.00378)
Supplement: Supplementary file 5 [file Table_4.PDF]

Supplementary Table S4. Pairwise comparison of the genetic distances based on nrITS sequences of *V. officinalis (14)* and *V. chamaedrys (9)* using Taxon DNA/SpeciesIdentifier v 1.7.8

**Distribution of all intraspecific distances**			
**Distances**	**Freq.**	**Perc.**	**Cumulative**
= 0.0%	61	53.5	46.5
0.0% to 0.5%	33	28.9	17.5
0.5% to 1.0%	13	11.4	6.1
1.0% to 1.5%	7	6.14	0.0
1.5% to 20%	0	0.0	0.0
> 20.0%	0	0.0	0.0

**Distribution of all interspecific distances**			
**Distances**	**Freq.**	**Perc.**	**Cumulative**
= 0.0%	0	0.0	0.0
0.0% to 1.0%	0	0.0	0.0
1.0% to 17%	0	0.0	0.0
18.0% to 19.0%	9	7.7	9.4
19.0% to 20.0%	39	33.3	42.7
20.0% to 21.0%	66	56.4	99.1
21.0% to 22.0%	1	0.9	100.0
> 22.0%	0	0.0	100.0
