# Supplementary material for: Veronica officinalis Product Authentication Using DNA Metabarcoding and HPLC-MS Reveals Widespread Adulteration with Veronica chamaedrys
Source: Front Pharmacol. 2017 Jun 19;8:378. doi: 10.3389/fphar.2017.00378 (PMC5474480; doi:10.3389/fphar.2017.00378)
Supplement: Supplementary file 6 [file Table_5.PDF]

Supplementary Table S5. Extracted DNA concentrations per sample and nrITS1 and nrITS2 amplicon concentrations as measured on a Fragment Analyzer (FA) in ng/ul

Product type	Product no.	Total DNA concentration (ng/ul)	nrITS1 - Amplicon concentration (ng/ul)	nrITS2 - Amplicon concentration (ng/ul)
Extract	1	0.063*	11.72	4.33
Extract	2	0.073*	5.21	9.13
Capsules	3	2.47	2.26	7.72
Herbal tea	4	28.45	1.69	6.76
Herbal tea	5	13.49	5.94	0.68
Herbal tea	6	28.56	12.65	14.07
Herbal tea	7	8.07	6.39	14.3
Herbal tea	8	84.93	8.007	11.04
Herbal tea	9	87.32	8.24	23.85
Herbal tea	10	3.97	16.83	17.22
Herbal tea	11	4.34	7.43	15.08
Herbal tea	12	9.09	18.46	14.64
Herbal tea	13	9.95	10.071	1.002
Candy	14	< 0.0005*	0.01	0.0003
Herbal tea	15	17.57	5.18	22.29
Herbal tea	16	9.98	23.13	0.078

*DNA concentration measured on Qubit, after 5X concentration by solvent vacuum evaporation
